# Association between non-cholesterol sterol concentrations and Achilles tendon thickness in patients with genetic familial hypercholesterolemia

**DOI:** 10.1186/s12967-018-1380-3

**Published:** 2018-01-15

**Authors:** Lucía Baila-Rueda, Itziar Lamiquiz-Moneo, Estíbaliz Jarauta, Rocío Mateo-Gallego, Sofía Perez-Calahorra, Victoria Marco-Benedí, Ana M. Bea, Ana Cenarro, Fernando Civeira

**Affiliations:** Unidad Clínica y de Investigación en Lípidos y Arteriosclerosis, Hospital Universitario Miguel Servet, Instituto de Investigación Sanitaria Aragón (IIS Aragón), CIBERCV, Universidad de Zaragoza, Av. Isabel La Católica, 1-3, 50009 Saragossa, Spain

**Keywords:** Non-cholesterol sterols, Xanthomas, Familial hypercholesterolemia

## Abstract

**Background:**

Familial hypercholesterolemia (FH) is a genetic disorder that result in abnormally high low-density lipoprotein cholesterol levels, markedly increased risk of coronary heart disease (CHD) and tendon xanthomas (TX). However, the clinical expression is highly variable. TX are present in other metabolic diseases that associate increased sterol concentration. If non-cholesterol sterols are involved in the development of TX in FH has not been analyzed.

**Methods:**

Clinical and biochemical characteristics, non-cholesterol sterols concentrations and Aquilles tendon thickness were determined in subjects with genetic FH with (n = 63) and without (n = 40) TX. Student-t test o Mann–Whitney test were used accordingly. Categorical variables were compared using a Chi square test. ANOVA and Kruskal–Wallis tests were performed to multiple independent variables comparison. Post hoc adjusted comparisons were performed with Bonferroni correction when applicable. Correlations of parameters in selected groups were calculated applying the non-parametric Spearman correlation procedure. To identify variables associated with Achilles tendon thickness changes, multiple linear regression were applied.

**Results:**

Patients with TX presented higher concentrations of non-cholesterol sterols in plasma than patients without xanthomas (*P* = 0.006 and 0.034, respectively). Furthermore, there was a significant association between 5α-cholestanol, β-sitosterol, desmosterol, 24S-hydroxycholesterol and 27-hydroxycholesterol concentrations and Achilles tendon thickness (*p* = 0.002, 0.012, 0.020, 0.045 and 0.040, respectively).

**Conclusions:**

Our results indicate that non-cholesterol sterol concentrations are associated with the presence of TX. Since cholesterol and non-cholesterol sterols are present in the same lipoproteins, further studies would be needed to elucidate their potential role in the development of TX.

**Electronic supplementary material:**

The online version of this article (10.1186/s12967-018-1380-3) contains supplementary material, which is available to authorized users.

## Background

Familial hypercholesterolemia (FH), among the most common inherited metabolic disorders, is due to a group of genetic disorders that result in abnormally high low-density lipoprotein (LDL) cholesterol levels that cause atherosclerotic plaque deposition in arteries and a markedly increased risk of coronary heart disease (CHD) at a young age [1, 2]. FH has an autosomal codominant inheritance, with homozygotes having twice the LDL cholesterol levels of heterozygotes [1, 2]. The frequency of the heterozygous FH (HeFH) state has traditionally been estimated at 1 in 500 and of the homozygous FH (HoFH) state at 1 in 1,000,000, although recent population analysis have estimated a prevalence as high as 1 in 250 [3]. In HeFH patients, the clinical expression is highly variable in terms of the severity of hypercholesterolemia, the presence of tendon xanthomas (TX), and the age of onset and severity of CHD, even in subjects sharing the same *LDLR* gene defect [4]. Patients with FH predominantly have an excess of CHD rather than cerebral or peripheral arterial disease. The risk of premature CHD is elevated to about 20-fold in HeFH in comparison with the general population, with the highest risk being noted in young untreated men. HoFH typically develop CHD by the second decade of life, but CHD deaths in the first decade of life have been also reported [5].

TX are lipid deposits within certain tendons, mainly Achilles and extensors of the hands, that produce diffuse and/or focal thickening and predispose to inflammation, occasionally causing pain and impairment of function [6]. TX in the presence of severe hypercholesterolemia with autosomal dominant transmission is highly specific of FH [2], and a mutation in one LDL receptor-related gene is commonly found in this scenario [7]. HoFH typically develops TX in the first decade of life, while TXs begin to appear after the third decade of life in 20–50% of affected HeFH [2]. It is unknown why some HeFH subjects develop TX and others do not, even sharing the same pathogenic LDLR mutation [4], and it may be related to interindividual variability in the inflammatory response of macrophages to oxidized LDL particles [8].

The presence of TX has important clinical implications, because it is a major criterion for the clinical diagnosis of HoFH [9] and HeFH [3], and more importantly, subjects with TX associate higher LDL cholesterol, more intense subclinical atherosclerosis and higher risk of CHD and, probably, deserve a more intense lipid lowering therapy [3, 10].

Macrophage-derived foam cells due to intracellular accumulation of cholesterol, extracellular cholesterol deposits and connective tissue are the main components of TX [11]. Hence, pathological characteristics of TX are similar to atherosclerotic vascular lesions, both situations being produced by an accumulation of intra and extracellular cholesterol and an inflammatory reaction surrounded by fibrosis tissue. Furthermore, major risk factors for the development of CHD are also associated with the presence of TX, including age, male gender or high LDL cholesterol [12]. These results suggest that xanthomas and coronary atherosclerosis may share etiology, explaining the association between TX and CHD in FH.

TXs are also present in other sterol metabolism disorders, such as sitosterolemia and cerebrotendinous xanthomatosis. Sitosterolemia is characterized by elevated phytosterol concentrations (β-sitosterol, campesterol…) and cerebrotendinous xanthomatosis by 27-hydroxycholesterol deficiency and accumulation of 5α-cholestanol [13, 14]. In both diseases, TX are the result of non-cholesterol sterols deposition rather than cholesterol, and, in both cases, TX can develop in absence of hypercholesterolemia, indicating that an increased exposure to non-cholesterol sterols, even at a plasma concentration lower than cholesterol, can induce sterol accumulation within macrophages [13, 14]. Considering than non-cholesterol sterol concentration is elevated in FH although with large interindividual variability [15], we hypothesized that the presence of TX in HeFH could be favored by elevated non-cholesterol sterol concentrations in plasma. If this is the case, then elevated non-cholesterol sterol concentrations could be involved in the production of TX in FH.

## Methods

### Study population

Hypercholesterolemic subjects from the Lipid Clinic at Hospital Universitario Miguel Servet, Zaragoza, Spain were selected. They were unrelated adults 18–79 years of age with the clinical and genetic diagnosis of FH (n = 103). FH was defined in presence of LDL cholesterol above the 95th percentile of the Spanish population [16], triglycerides below 200 mg/dl, familial presentation (at least one first-degree relative with the same phenotype) and heterozygous for a functional mutation in *LDLR*, *APOB, PCSK9 or APOE* genes. Exclusion criteria: secondary causes of hypercholesterolemia including: severe obesity (body mass index (BMI) > 35 kg/m^2^), poorly controlled type 2 diabetes (HbA1c > 8%), renal disease with glomerular filtration rate < 30 ml/min and/or macroalbuminuria, liver diseases (ALT > 3 times upper normal limit), hypothyroidism (TSH > 6 mIU/l), pregnancy, autoimmune diseases and protease inhibitors consumption. Subjects disclosing *APOE* ε2/ε2 genotype were also excluded for this study. Subjects with previous CVD were excluded except if they were not on lipid-lowering drugs.

CVD risk factors assessment, personal and family history of CVD, consumption of drugs affecting intestinal or lipid metabolism and anthropometric measurements were performed in all participants. All subjects signed informed consent to a protocol previously approved by our local ethical committee (Comité Ético de Investigación Clínica de Aragón, Zaragoza, Spain).

### Tendon xanthomas measurements

TXs were measured in the Achilles tendons using high resolution sonography. Standardized equipment and operating procedures were used for Achilles tendon thickness measurements as previously described [17]. The variables of interest were mean and maximum Achilles tendon thickness, bilaterally. TX was defined when Achilles tendon maximum thickness was over 5.3 and 5.7 mm in men < 45 and > 45 years, and over 4.8 and 4.9 mm in women < 50 and > 50 years, respectively. These thickness thresholds have demonstrated to be good discriminators for the TX diagnosis in HeFH [17].

### Clinical and laboratory parameters

Fasting blood for biochemical profiles was drawn after at least 5–6 weeks without hypolipidemic drug treatment, plant sterols or fish oil supplements. Cholesterol and triglycerides were determined by standard enzymatic methods. High density lipoprotein (HDL) cholesterol was measured by a precipitation technique. Apolipoprotein (apo) A1, apo B, lipoprotein(a) (Lp(a)) and C-reactive protein were determined by inmunonephelometry using IMMAGE-Immunochemistry System (Beckman Coulter).

DNA was isolated from EDTA blood samples following standard protocols. *APOE* sequencing was performed in all study subjects as previously described [18]. The screening for *LDLR*, *APOB* and *PCSK9* mutations was carried out using Lipochip Platform (Progenika Biopharma S. A., Bilbao, Spain). The platform consists of two consecutive steps: the first one is the LIPOchip1 microarray analysis for the detection of the most frequent Spanish point mutations in the *LDLR* gene and in the *APOB* exon 26, as well as CNVs in *LDLR*. When the LIPOchip1 microarray gives a negative result (no mutation is found), the *LDLR*, *APOB* (binding domain) and *PCSK9* gene coding sequences, exon–intron boundaries, and short proximal intronic sequences were sequenced with a GS Junior system (Roche Diagnostics Corporation, Basel, Switzerland) [19].

### Serum sterol determinations

Serum concentration of 5α-cholestanol, β-sitosterol, campesterol, stigmasterol, sitostanol, desmosterol, lanosterol, 24S-hydroxycholesterol, 27-hydroxycholesterol and 7α-hydroxy-4-cholesten-3-ona and cholesterol were quantified after 10 h of fasting. They were quantified using high performance liquid chromatography tandem mass spectrometry (HPLC–MS/MS) according to the method previously described [20], and were expressed as mg/dl as well as normalized to mg/dl of total cholesterol. Briefly, 100 µl of serum were transferred to a screw-capped vial and deuterium-labelled internal standard, (^2^H6) cholesterol-26,26,26,27,27,27, (7.9 mM), was added to determine non-cholesterol sterol concentrations. Another 100 µl of serum were transferred to a screw-capped vial, deuterium-labelled internal standard, (^2^H7) cholesterol-25,26,26,26,27,27,27, was added to determine cholesterol. Alkaline hydrolysis was performed for 20 min at 60 °C in an ultrasound bath and extracted twice with 3 ml of hexane. The extracts were loaded onto the SPE cartridge (1 mg, Discovery DSC-18, Supelco, Spain) which was preconditioned with 400 µl of methanol and gravity eluted. Sterols were desorbed with 1.4 ml of 2-propanol by gravity and 40 µl of the final mixtures were injected into the HPLC–MS/MS system.

### Statistical analysis

Statistical analysis was performed using SPSS software version 15.0 (Chicago, Illinois, USA) using a significance level of *P* values < 0.05. Data are expressed as mean ± standard deviation for continuous variables with normal distribution and medians (percentile 25–percentile 75) for variables with a skewed distribution. Student t test o Mann–Whitney test were used accordingly. Categorical variables were compared using a Chi square test. ANOVA and Kruskal–Wallis tests were performed to multiple independent variables comparison. Post hoc adjusted comparisons were performed with Bonferroni correction when applicable. Correlations of parameters in selected groups were calculated applying the non-parametric Spearman correlation procedure. To identify variables associated with Achilles tendon thickness changes, we applied multiple linear regression with age, height and 5α-cholestanol, β-sitosterol, campesterol, stigmasterol, sitostanol, desmosterol, lanosterol, 24S-hydroxycholesterol, 27-hydroxycholesterol and 7α-hydroxy-4-cholesten-3-ona as independent variables.

## Results

### Clinical and biochemical characteristics

The main clinical and biochemical characteristics of 103 FH subjects without and with xanthomas are presented in Table 1. Both groups did not have statistically significant differences in most of the clinical and biochemical characteristics. Corneal arcus was significantly higher in patients with xanthomas than in those patients without xanthomas (*P* < 0.009*).* No differences in age, tobacco consumption, CVD, diabetes, hypertension neither other characteristics were found between patients without and with xanthomas.

**Table 1:** Clinical and biochemical characteristics of study subjects without or with tendon xanthomas

	FH n = 103		
	Subjects without xanthomas n = 63	Subjects with xanthomas n = 40	*p*
Age, years	39.4 ± 13.2	40.7 ± 13.0	0.639
Men, n (%)	32 (50.8)	18 (45.0)	0.566
Current smokers, n (%)	19 (31.7)	7 (17.5)	0.286
Non-smokers, n (%)	30 (50.0)	24 (60)	
Former smokers, n (%)	11 (18.3)	9 (22.5)	
Carotid plaque, n (%)	9 (15.5)	5 (13.2)	0.674
Diabetes, n (%)	2 (3.2)	1 (2.5)	0.843
Hypertension, n (%)	7 (11.1)	3 (7.5)	0.546
Body mass index, Kg/m^2^	24.2 ± 3.7	25.0 ± 3.4	0.251
Corneal arcus, n (%)	11 (17.5)	23 (41.0)	0.009
Lipoprotein(a), mg/dl	27.6 (12.4–45.5)	26.0 (11.2–51.6)	0.994
Glucose, mg/dl	87 (81–93)	86 (80–95)	0.935
C-reactive protein, g/l	1.8 (0.5–3.4)	0.85 (0.52–2.1)	0.202
*APOE* genotype E3/3, n (%)	38 (60.3)	27 (69.2)	0.736
*APOE* genotype E3/2, n (%)	7 (11.1)	4 (10.3)	
*APOE* genotype E3/4, n (%)	17 (27.0)	7 (17.9)	
*APOE* genotype E2/4, n (%)	1 (1.6)	1 (2.6)	

Numerical variables with normal distribution are expressed as mean ± standard deviation and those with skewed distribution are expressed as median [percentile 25–percentile 75]. t Student or Mann–Whitney or Chi square tests were used as appropriate

### Lipid profile, Achilles tendon thickness and serum non-cholesterol sterols

The lipid profile and Achilles tendon thickness of FH without and with xanthomas is presented in Table 2. Patients with xanthomas had significantly higher values of total cholesterol, non-HDL cholesterol, LDL cholesterol and apo B than patients without xanthomas. No differences in triglycerides, HDL cholesterol and apo A1 were found between both studied groups of patients. As expected, the maximum and the mean Achilles tendon thickness were significantly higher in FH subjects with xanthomas than in those FH subjects without xanthomas.

**Table 2:** Lipid profile, non-cholesterol sterols and Achilles tendon thickness of subjects without or with xanthomas

	FH		
	Subjects without xanthomas n = 63	Subjects with xanthomas n = 40	*p*
Total cholesterol, mg/dl	308.8 ± 48.2	354.9 ± 63.1	< 0.001
Triglycerides, mg/dl	93 (66–149)	98 (65–175)	0.707
HDL cholesterol, mg/dl	51.9 ± 15.7	54.9 ± 16.4	0.369
Non-HDL cholesterol, mg/dl	256.8 ± 47.3	300.1 ± 62.0	< 0.001
LDL cholesterol, mg/dl	235.4 ± 51.6	274 ± 55.0	0.001
Apolipoprotein A1, mg/dl	148.6 ± 34.8	143.4 ± 31.8	0.450
Apolipoprotein B, mg/dl	172.5 ± 31.4	191.4 ± 48.1	0.019
Maximum Achilles tendon thickness, mm	4.70 (4.43–4.95)	5.71 (5.30–6.88)	< 0.001
Mean Achilles tendon thickness, mm	4.51 (4.16–4.73)	5.49 (5.05–6.63)	< 0.001
Cholesterol HPLC–MS/MS, mg/dl	314.6 ± 52.1	351.4 ± 73.1	0.004
5α-cholestanol, mg/dl	0.665 ± 0.269	0.843 ± 0.375	0.006
β-sitosterol, mg/dl	0.602 ± 0.319	0.767 ± 0.462	0.034
Campesterol, mg/dl	0.329 ± 0.173	0.384 ± 0.230	0.176
Stigmasterol, mg/dl	0.0462 ± 0.0296	0.0528 ± 0.0287	0.269
Sitostanol, mg/dl	0.0217 (0.0093–0.0551)	0.0338 (0.0119–0.0565)	0.614
Desmosterol, mg/dl	0.773 ± 0.345	0.896 ± 0.430	0.109
Lanosterol, mg/dl	0.820 ± 0.383	0.896 ± 0.430	0.566
24S-hydroxycholesterol, mg/dl	0.0100 ± 0.0042	0.0115 ± 0.0061	0.142
27-hydroxycholesterol, mg/dl	0.0163 (0.0117–0.0210)	0.0167 (0.0123–0.0270)	0.161
7α-hydroxy-4-cholesten-3-ona, mg/dl × 10	0.0370 (0.0253–0.0542)	0.0358 (0.0242–0.0511)	0.735

Values are mean ± SD or median (interquartile range). *P* refers to differences calculated by Student’s t test for data normally distributed and Mann–Whitney U test for skewed data

LDL denotes low density lipoprotein; HDL, high density lipoprotein; HPLC–MS/MS, high performance liquid chromatography tandem mass spectrometry

Non-cholesterol sterols values are also presented in Table 2. 5α-cholestanol and β-sitosterol, cholesterol absorption surrogate markers, were statistically significant higher in FH subjects with xanthomas than in FH subjects without xanthomas (*P* = 0.006 and 0.034, respectively). No differences in cholesterol synthesis surrogate markers neither oxysterols were found between FH subjects with and without xanthomas.

### Achilles tendon thickness association with serum non-cholesterol sterols concentrations

**Fig. 1:**
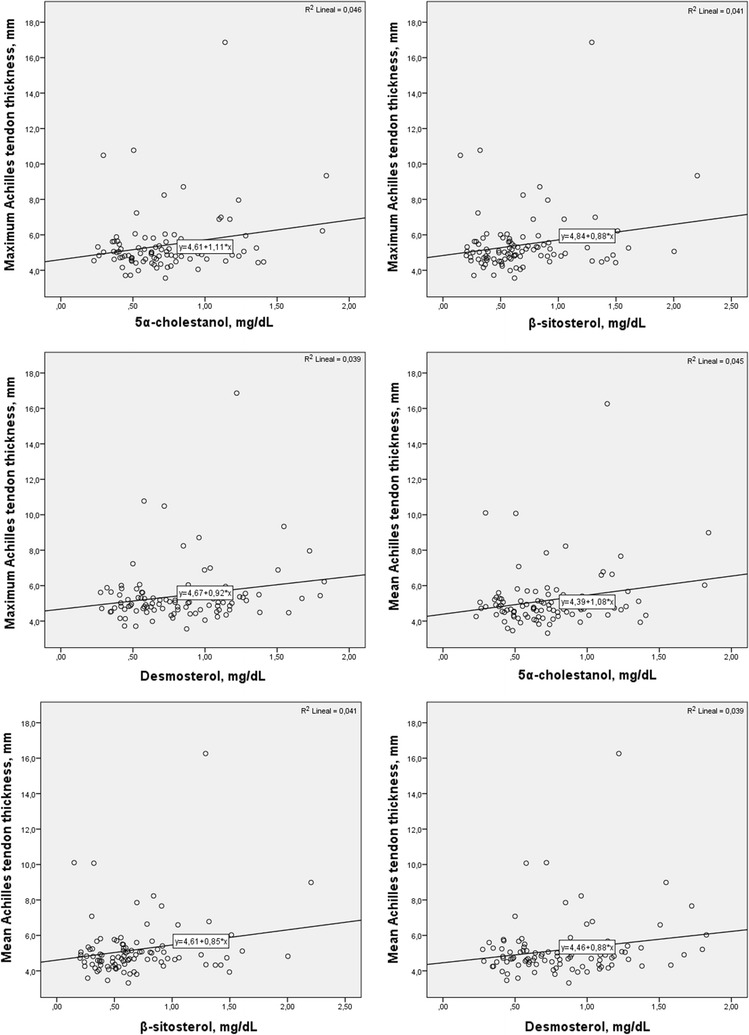
Association between serum non-cholesterol sterols and the maximum and the mean Achilles tendon thickness in subjects with familial hypercholesteroemia

Linear regression analysis showed a significant positive association of 5α-cholestanol and β-sitosterol with maximum and mean Achilles tendon thickness. 5α-cholestanol and β-sitosterol concentration explained 23.2 and 23.1% of the maximum Achilles tendon thickness, and 20.3 and 20.4% of the mean Achilles tendon thickness variation, respectively (Table 3). Desmosterol concentration also showed a significant positive association with the maximum and the mean Achilles tendon thickness. Desmosterol concentration determined 19.5% of maximum and 19.6% of mean Achilles tendon thickness (Table 3). 24S-hydroxycholesterol and 27-hydroxycholesterol concentrations also showed a significant positive association with the maximum and the mean Achilles tendon thickness. 24S-hydroxycholesterol concentration determined 18.3% of maximum Achilles tendon thickness and 27-hydroxycholesterol concentration determined 18.5% of maximum and 18.5% of mean Achilles tendon thickness (Table 3). The rest of the measured non-cholesterol sterols were not significantly associated to the maximum neither the mean Achilles tendon thickness. The positive associations between 5α-cholestanol, β-sitosterol and desmosterol and the maximum and the mean Achilles tendon thickness are shown in the Fig. 1. However, the significant association of non-cholesterol sterols with Achilles tendon thickness disappeared when non-cholesterol sterols concentrations were adjusted by total cholesterol and LDLc concentrations (Additional file 1: Tables S1, S2). There was a positive correlation between total cholesterol and LDLc levels and maximum and mean Achilles tendon thickness (R_TC_ = 0.302; *p* = 0.003, R_LDLc_ = 0.304; *p* = 0.003 and R_TC_ = 0.302; *p* = 0.001, R_LDLc_ = 0.336; *p* = 0.0001, respectively).

**Table 3:** Achilles tendon thickness association with serum non-cholesterol sterols concentrations

	FH n = 103				
	Achilles tendon thickness	B	[95% CI]	*p*	R^2^
5α-cholestanol, mg/Dl	Maximum	− 0.033	− 0.054, − 0.013	0.002	23.2
	Mean	− 0.035	− 0.057, − 0.012	0.003	23.1
β-sitosterol, mg/dl	Maximum	− 0.022	− 0.040, − 0.005	0.012	20.3
	Mean	− 0.024	− 0.232, − 2.489	0.015	20.4
Campesterol, mg/dl	Maximum	− 0.031	− 0.067, 0.004	0.081	18.6
	Mean	− 0.033	− 0.071, 0.005	0.088	18.9
Stigmasterol, mg/dl	Maximum	− 0.172	− 0.412, 0.068	0.157	16.5
	Mean	− 0.191	− 0.449, 0.066	0.144	17.0
Sitostanol, mg/dl	Maximum	0.037	− 0.197, 0.270	0.756	14.7
	Mean	0.019	− 0.232, 0.270	0.881	15.1
Desmosterol, mg/dl	Maximum	− 0.022	− 0.041, − 0.004	0.020	19.5
	Mean	− 0.023	− 0.043, − 0.003	0.025	19.6
Lanosterol, mg/dl	Maximum	− 0.100	− 0.444, 0.245	0.567	14.9
	Mean	− 0.110	− 0.480, 0.260	0.556	15.4
24S-hydroxycholesterol, mg/dl	Maximum	− 1.420	− 2.810, − 0.030	0.045	18.3
	Mean	− 1.428	− 2.926, 0.070	0.061	18.3
27-hydroxycholesterol, mg/dl	Maximum	− 0.820	− 1.600, − 0.04	0.040	18.5
	Mean	− 0.858	− 1.697, − 0.018	0.045	18.7
7α-hydroxy-4-cholesten-3-ona, mg/dl × 10	Maximum	0.339	− 1.526, 2.203	0.719	14.7
	Mean	0.425	− 1.579, 2.428	0.675	15.2

The linear regression analysis was adjusted by age and height

## Discussion

The mechanism of production of TX in FH is not known. Traditionally, it has been observed that the appearance of xanthomas is related to the appearance of classic cardiovascular risk factors and to the concentration of LDL cholesterol [2]. However, these factors do not account for the majority of cases with xanthomas. Sometimes, xanthomas are clustered in families; therefore, it could be thought that the mutation responsible for hypercholesterolemia would play a role in their development. However, studies performed in populations where a single mutation is prevalent, such as the Canadians in Quebec region, show that although they share a mutation, they still maintain a huge variability in the presentation of xanthomas [4]. This would indicate that other genetic and/or environmental factors that are shared in families could play a role in the development of xanthomas.

The concentration of serum non-cholesterol sterols is highly variable and has an evident polygenic component, as we have recently been able to demonstrate in families with hypercholesterolemia [21]. This indicates that both the absorption and the synthesis of cholesterol have some genetic control and could be involved in the familial association of TX.

However, our hypothesis has not been fully confirmed in this study. Although non-cholesterol sterols, especially markers of intestinal absorption and hepatic synthesis, are elevated in subjects with xanthomas, this association disappears when adjusted for total cholesterol concentration. Non-cholesterol sterols, like cholesterol, are transported in lipoproteins, especially within the LDL particles; therefore in the FH, where the number of LDL particles is very high, the transport of sterols is expected to be increased [22]. Hence, most authors recommend adjusting the values of non-cholesterol sterols by the concentration of total cholesterol [23]. Since cholesterol and non-cholesterol sterols are bound in the same lipoprotein particles, it is not possible in our study to differentiate the cholesterol-dependent effect of the sterol-dependent effect. The fact that different diseases that associate increase in the sterol concentrations develop TX indicates that non-cholesterol sterols by themselves, even in the absence of hypercholesterolemia, are able to promote TX [13, 14].

The associated inflammatory tissue in xanthoma is very similar to that of the atheroma plaque, predominantly lipid-laden macrophages, especially cholesterol esters [24]. The modified LDL particle captured by macrophages from both the wall and the xanthomas captures both cholesterol and sterols. However, the capacity of esterification by macrophages is much higher for cholesterol than for sterols that accumulate as free sterols in the lesions [25]. If this differential mechanism is the responsible of the appearance of xanthomas is not well known, but what has been well demonstrated is that the inflammatory reaction induced by LDL is different from some subjects to others [26]. In a previous study, we had demonstrated that macrophages from FH subjects with TX stimulated with oxLDL showed an overexpression of chemokine IL-8 and CXCL3 genes and produced higher IL-8 protein levels than macrophages from FH subjects without TX. However, we could not demonstrate if the observed inflammatory effect was LDL cholesterol dependent [27, 28].

The use of plant sterols in the treatment of hypercholesterolemia is a well-accepted cholesterol reduction procedure that has also been proposed as a lipid-lowering treatment in HeHF [29]. However, as far as we know, the use of plant sterols in familial hypercholesterolemia has not been associated with the development of xanthomas. The importance of the sterol 27-hydroxylase-mediated mechanism in the xanthoma development is illustrated by the fact that patients who lack this enzyme develop xanthomas and premature atherosclerosis in spite of normal levels of circulating cholesterol [30]. Patients with the inherited disease cerebrotendinous xanthomatosis lack the enzyme sterol 27-hydroxylase (CYP27A1). In humans without this enzyme defect, 27-hydroxycholesterol accumulates in atherosclerotic lesions. Furthermore, the enzyme sterol 27-hydroxylase has been identified in tendocytes and macrophages and induces conversion of cholesterol to the more polar metabolite 27-hydroxycholesterol, which can be eliminated from the cells more effectively [31].

Our study has several limitations. First, we do not know the concentration of non-cholesterol sterols in Achilles tendon, we have only measured them in serum and it would have been interesting to know the real concentration of sterols in xanthomas instead of in blood. Secondly, the lipid-lowering treatment modifies the development of xanthomas and the subjects of our study, although they were untreated at the time of analysis, most of them had variable time with statins that may have altered tendon thickness. Finally, the definition of TX is arbitrary and based on a continuous variable, such as tendon thickness; if other definition of TX could yield different results would be possible, although seems improbable because those subjects in the highest quartile of Achilles tendon thickness did not significantly differ from the rest of quartiles.

## Conclusion

HeFH subjects with TX present higher concentrations of non-cholesterol sterols in serum than HeFH subjects without TX. Furthermore, there is a significant association between 5α-cholestanol and β-sitosterol serum concentration and Achilles tendon thickness. Our results indicate that non-cholesterol sterol concentrations are associated with the presence of xanthomas in familial hypercholesterolemia. Since cholesterol and non-cholesterol sterols are present in the same lipoprotein particles further studies would be needed to elucidate their role in the development of TX.

## Additional file

**Additional file 1: Table S1.** Achilles tendon thickness association with serum non-cholesterol sterols concentrations adjusted by LDLc. **Table S2.** Achilles tendon thickness association with serum non-cholesterol sterols concentrations adjusted by total cholesterol.
