# Supplementary material for: Association between non-cholesterol sterol concentrations and Achilles tendon thickness in patients with genetic familial hypercholesterolemia
Source: J Transl Med. 2018 Jan 15;16:6. doi: 10.1186/s12967-018-1380-3 (PMC5769342; doi:10.1186/s12967-018-1380-3)
Supplement: Supplementary file 1 — Additional file 1: Table S1. Achilles tendon thickness association with serum non-cholesterol sterols concentrations adjusted by LDLc. Table S2. Achilles tendon thickness association with serum non-cholesterol sterols concentrations adjusted by total cholesterol. [file 12967_2018_1380_MOESM1_ESM.docx]

**Supplementary Tables**

**Supplementary Table 1. Achilles tendon thickness association with serum non-cholesterol sterols concentrations adjusted by LDLc.**

|  | **FH**  n=103 | | | | |
| --- | --- | --- | --- | --- | --- |
|  | **Achilles tendon thickness** | **B** | **[95% CI]** | ***p*** | **R^2^** |
| 5α-cholestanol | Maximum | -0.016 | -0.039,0.006 | 0.157 | 24.9 |
|  | Mean | -0.018 | -0.043,0.007 | 0.152 | 23.6 |
| β-sitosterol | Maximum | -0.012 | -0.029,0.006 | 0.191 | 24.6 |
|  | Mean | -0.013 | -0.032,0.006 | 0.184 | 23.3 |
| Desmosterol | Maximum | -0.006 | -0.026,0.014 | 0.529 | 23.5 |
|  | Mean | -0.007 | -0.029,0.014 | 0.512 | 22.2 |
| 24S-hydroxycholesterol | Maximum | -0.412 | -1.844,1.019 | 0.569 | 23.5 |
|  | Mean | -0.396 | -1.954,1.161 | 0.614 | 22.0 |
| 27-hydroxycholesterol | Maximum | -0.116 | -0.944,0.711 | 0.780 | 23.2 |
|  | Mean | -0.156 | -1.056,0.743 | 0.731 | 21.9 |

The linear regression analysis was adjusted by age and height.

**Supplementary Table 2. Achilles tendon thickness association with serum non-cholesterol sterols concentrations adjusted by total cholesterol.**

|  | **FH**  n=103 | | | | |
| --- | --- | --- | --- | --- | --- |
|  | **Achilles tendon thickness** | **B** | **[95% CI]** | ***p*** | **R^2^** |
| 5α-cholestanol | Maximum | -0.016 | -0.038,0.007 | 0.164 | 28.1 |
|  | Mean | -0.018 | -0.042,0.007 | 0.157 | 26.6 |
| β-sitosterol | Maximum | -0.011 | -0.028,0.007 | 0.233 | 27.7 |
|  | Mean | -0.012 | -0.031,0.007 | 0.223 | 26.2 |
| Desmosterol | Maximum | -0.006 | -0.026,0.013 | 0.525 | 26.9 |
|  | Mean | -0.007 | -0.028,0.014 | 0.498 | 25.4 |
| 24S-hydroxycholesterol | Maximum | -0.464 | -1.846,0.918 | 0.507 | 26.9 |
|  | Mean | -0.467 | -1.973,1.039 | 0.539 | 25.3 |
| 27-hydroxycholesterol | Maximum | -0.123 | -0.937,0.691 | 0.765 | 26.6 |
|  | Mean | -0.165 | -1.052,0.721 | 0.712 | 25.1 |

The linear regression analysis was adjusted by age and height.
